# Generic Switching and Non-Persistence among Medicine Users: A Combined Population-Based Questionnaire and Register Study

**DOI:** 10.1371/journal.pone.0119688

**Published:** 2015-03-16

**Authors:** Jette Rathe, Morten Andersen, Dorte Ejg Jarbøl, René dePont Christensen, Jesper Hallas, Jens Søndergaard

**Affiliations:** 1 Research Unit of General Practice, Institute of Public Health, University of Southern Denmark, Odense, Denmark; 2 Centre for Pharmacoepidemiology, Karolinska Institutet, Department of Medicine Solna, Stockholm, Sweden; 3 Research Unit of Clinical Pharmacology, Institute of Public Health, University of Southern Denmark, Odense, Denmark; Carl von Ossietzky University of Oldenburg, GERMANY

## Abstract

**Background:**

Generic substitution means that one medicinal product is replaced by another product containing the same active substance. It is strictly regulated with respect to its bioequivalence, and all products must have undergone appropriate studies. Although generic substitution is widely implemented, it still remains to be answered how generic switch influences persistence to long-term treatment, and if it is modified by patients’ concerns about medicine and views on generic medicine. This study focuses on users of antidepressants and antiepileptics, and their experience of generic switching.

**Methods:**

The study was an observational cohort study. By use of a prescription database, we identified patients who had redeemed prescriptions on generically substitutable drugs, and a questionnaire was mailed to them. We analyzed predictors of discontinuation in relation to generic switch and patients’ attitudes towards generic medicines and concerns about their medicine.

**Results:**

Patients who experience their first-time switch of a specific drug were at higher risk of non-persistence, Hazard Ratio 2.98, 95% CI (1.81;4.89) versus those who have never switched, and 35.7% became non-persistent during the first year of follow-up. Generic switching did not influence persistence considerably in those having previous experience with generic switching of the specific drug. Stratified analyses on users of antidepressants and antiepileptics underpin the results, showing higher risk of non-persistence for first-time switchers for both drug categories.

**Conclusion:**

In conclusion, patients who are first-time switchers of a specific drug were at higher risk of non-persistence compared to never switchers and those having experienced previous generic switching.

## Background

Although generic substitution is widely implemented, it still remains to be answered how the substitution influences persistence to long-term treatment and if it is modified by patients’ concerns about medicine and views on generic medicine.

Generic substitution means that one medicinal product is replaced by another product containing the same active substance, and generic substitution is implemented in many countries worldwide. In some countries generic substitution is only regarding a switch from brand name to generic drug, while in Denmark generic substitution includes all types of switches between drugs containing the same active substance [1,2]. It is strictly regulated with respect to its bioequivalence, and all products must have undergone appropriate studies [3]. However, generic substitution has always been accompanied with concerns about clinical equivalence in terms of safety and effectiveness, and whether it may have important consequences for the medicine users’ adherence [4,5]. Research on the subject often focuses on one shift from a manufacturer’s drug to a generic drug or on incident users whose prescription is substituted at their first redemption [6–8]. Most of these studies did not identify significant associations between generic substitution and non-adherence [6,9,10], but one study assessing the association between generic substitution and persistence showed reduced persistence [7]. So far, studies of the effect of generic drug substitution on drug continuation have not focused on patients overall experience of generic switches.

Studies on patients’ beliefs about medicine have shown associations with patients’ adherence towards drug treatment. Those with stronger beliefs about medicine as being harmful and with concerns about treatment were less adherent, while patients with stronger perceptions of necessity of treatment showed higher adherence [11,12]. How these beliefs about medicine influence patients’ persistence after generic switching has not been shown. Hence, we aimed to analyze, in a cohort study combining data from a register and questionnaires, predictors of discontinuation in relation to generic switch and patients’ attitudes towards generic medicines and concerns about their medicine.

## Methods

This study is part of a larger project on the consequences of generic substitution initiated by the Danish Ministry of Health comprising qualitative interviews, questionnaires and register data [13–15].

### Study design

By use of a prescription database, we identified patients who had redeemed prescriptions on generically substitutable drugs and analyzed predictors of discontinuation in relation to generic switch and patients’ attitudes towards generic medicines and concerns about their medicine.

The study was an observational cohort study comprising 4000 randomly selected patients. The patients were aged 20 years or older and living in the Region of Southern Denmark, who in September 2008 redeemed prescriptions with general reimbursement and where generic substitution was recommended.

A substitutable drug was defined as a medicinal product approved for generic substitution by the Danish regulatory authorities. In the Anatomical-Therapeutic-Chemical (ATC) system, substitutable medicines have the same 5^th^ level code corresponding to the active substance or a combination of substances [16].

### Setting

Generic substitution was implemented in Denmark in 1991 and represents 68% of the Danish drug consumption [17]. Pharmacies are obliged to substitute a generic version of a drug according to a defined list, if the general practitioner (GP) has not explicitly stated that it should not be performed, or the patient insists on having the more expensive drug. In both cases, the patients have to pay the price difference [18]. In Denmark patients use whichever pharmacy they wish to. Drug prices are regulated every 14 days and The National Health Service only reimburses the price of the least expensive product. The pharmacy proposes the cheapest drug within a limit of 5–20 Danish kroner’s (0.8–3.3 US$) price difference. A prescription always comprises the brand name, and prescription of the substance name is not allowed [17,19].

The Danish healthcare system is tax funded, providing free access to general practice, outpatient clinics and hospital care for all inhabitants irrespective of age, socioeconomic status and geographical residence. Reimbursement increases with patients’ expenses for prescription medication [20]. All Danish citizens are registered with a unique personal identification number, used in all national registers, thus enabling accurate record linkage [21–23].

### Data sources

The patients were identified by means of Odense PharmacoEpidemiologic Database (OPED) [24], covering the population in the Region of Southern Denmark (1.19 million). Only prescriptions issued by GPs were included. For each patient we focused on one purchase of a generically substitutable drug (index drug) during September 2008. OPED data were used to obtain information on the patients’ prescriptions on their day of inclusion (index date), as well as information on any other prescription during the preceding 12 months, including ATC code, brand name and date of purchase. We were thus able to identify drug switches likely to be due to generic substitution and the number of different drugs dispensed. A generic switch was defined as having taken place, if the patient had previously purchased the same pharmacological substance under a different name or by a different manufacturer. This distinction between drug products was based on brand name, registration holder (having the right to marketing) and importer or parallel importer. Our primary predictor was the generic switch of the index drug. A previous study from our group showed that experience with earlier generic switch within the index ATC code was clearly positive associated with the generic switch and was included in the model [14]. Thus we were able to distinguish between first-time generic switchers within the same ATC code and patients with previous experience. As a possible confounder we included the “number of different drugs” defined as the number of different ATC codes different from the index ATC code at the 5th level purchased by the patient during the 12 months prior to their index date. Number of different drugs was used as a proxy for comorbidity, as comorbidity may influence patients’ persistence and the choice of switching between generically substitutable drugs.

The variable “redemptions of the index drug within 1 year prior to index date” was used to illustrate patients’ experience with the index ATC code 365 days before the index date. Information on residency and vital status of the cohort member was retrieved from the demographic data in OPED [24].

### Study subjects and questionnaire

Our cohort comprises 2000 users of antiepileptics and 2000 users of antidepressants. Details of the sample selection are described in a prior publication [14]. Patients were eligible for inclusion if they had made at least one other purchase of the same drug or one of its generic alternatives within 120 days prior to their index date [13]. For each patient we focused on the purchase of one generically substitutable drug (index drug). To prevent vulnerable patients (e.g. patients with severe terminal disease or dementia) from receiving the questionnaire, the GPs were asked whether it was appropriate to approach their patients. This is standard procedure when using data from OPED to approach patients.

Questionnaires were mailed out in December 2008. A reminder was sent two weeks later. The questionnaire was adapted to the individual subject with reference to their specific drug (index drug) in every question and index date printed on the questionnaire. As a quality control the patient had to confirm purchase of the index drug to be included in the study.

The questionnaire included scales from the “Beliefs about Medicine Questionnaire” (BMQ) and ad hoc scales developed on the basis of a literature review and a qualitative interview study, which have been reported elsewhere [13]. The BMQ was translated into Danish by means of a standardized forward-backward translation [25] and finally approved by Rob Horne, who developed the questionnaire. The BMQ is a validated and widely used psychometric instrument, which assesses patients’ beliefs about medicines prescribed for personal use and beliefs about medicine in general [26]. The *specific concern* scale (from the BMQ) was used as measure of beliefs about the index drug. It consists of 6 items and assesses concerns about prescribed medication based on beliefs about the danger of dependence and long-term toxicity and the disruptive effects of medication, e.g. “it worries me that I have to take this medicine” [26,27]. The items in the BMQ scale were measured on a 5-point Likert response scale (strongly disagree to strongly agree).

Furthermore, we constructed the scale *Views on generic medicine* for this questionnaire. The scale was based on 4 items concerning side effects, quality and effectiveness of generic medicine. The items were also measured on a 5-point Likert response scale (1: strongly agree to 5: strongly disagree). We analyzed the internal consistency using Cronbach’s α. A value of 0.88 was found, suggesting that the scale is reliable.

A person’s score was calculated as the average of the non-missing scale items, if at least 60% of the scale items were answered. If less than 60% of the items were answered, the score was treated as missing. The BMQ subscale ranged between 1 and 5, and a high score meant a stronger belief in the concept described by the scale. The scale *Views on generic medicine* ranged from 1 to 5, and a low score meant a positive view on generic medicines.

The questionnaire was pilot tested prior to the survey focusing on comprehensibility, relevance, acceptability and feasibility. Interviews were carried out with 18 medicine users purchasing their drug at community pharmacies, and the interviews were discussed in an academic setting of healthcare researchers.

### Data analysis

Persistence represents the time over which a patient continues to fill a prescription, or the time from the initial filling of the prescription until the patient discontinues refilling of prescription [28]. In this study a subject was considered to be a medication user from the index date and for the subsequent number of days corresponding to the number of tablets of the prescription. A treatment episode was considered to have ended, if the interval between two prescriptions exceeded a period covered by the number of tablets prescribed plus a grace period of 90 days. We assume the patients as a minimum take 1 tablet per day. The grace period was introduced to allow for some degree of non-adherence and for irregular dispensing due to stockpiling. We defined non-persistence as the first episode during the study period when a subject failed to present a subsequent prescription within the time window defined by the duration of the preceding prescription [29,30].

To analyze associations between generic switching and non-persistence, we used a Cox proportional hazards model to calculate Hazard Ratios (HR) and corresponding 95% confidence intervals (CI) and Kaplan-Meier curve to show time to non-persistence [28]. The analysis period was defined from the index date and 365 days ahead. Non-persistence events were registered on the day the number of tablets expired. An event was classified as such if it took place within the 365 days of follow-up or before the patient moved out of the Region or died. Patients were censored on the day of death, date of moving or at the time the analysis period ended, if an event had not taken place. If censoring occurred during the grace period, the date of censoring was set to the day that the number of tablets expired.

In the Cox model we adjusted for potential confounders such as age, gender, number of different drugs, concerns about medicine and views on generics. Sensitivity analyses were made, assessing the influence of grace periods using 30 days, 60 days, 90 days and 120 days.

All analyses were performed using Stata Release 11.0 (Stata-Corp, College Station, TX, USA).

#### Ethics statement

Written informed consent was obtained by all participants for their clinical records to be used in this study. According to the Act on Biomedical Research Ethics Committee System, the project was not a biomedical research project and therefore did not need the Research Ethics Committee’s approval. The anonymity of patients was strictly preserved throughout the data entry and analysis process. The study was approved by the Danish Data Protection Agency (journal number 2008–41–2364)

## Results

**Fig 1:**
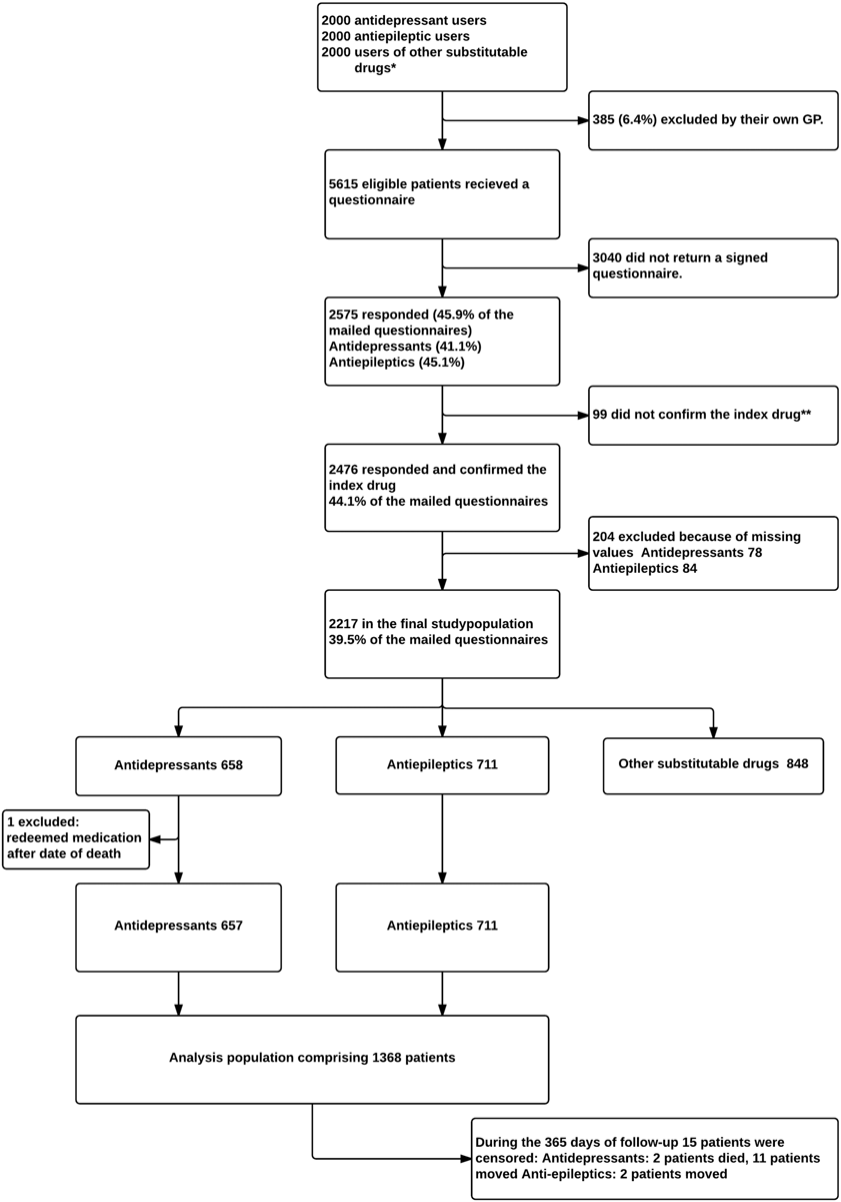
Flowchart of the postal survey process. Generic switch is defined as a purchase of a drug different from the patients’ previously purchased drug within the same ATC code. * “Other substitutable drugs”: consisted of a wide range of medicines considered as a random sample of what is used for long-term treatment; **Index drug: the purchase of a generically substitutable drug in September 2008 (index date); +GS: experienced a generic switch;-GS: did not experience a generic switch

A total of 2476 patients (44.1%) responded to the questionnaire and 1368 patients who used antiepileptics or antidepressants were included in the analysis (Fig. 1). During the analysis period 15 patients either moved out of the Region of Southern Denmark or died and were therefore censored. Table 1 shows the baseline characteristics according to whether the patients had experienced a generic switching stratified on drug categories.

**Table 1:** Characteristics of the study population, stratified on whether a generic switch took place on the index day.

Characteristics *(N = 1368)*	Antiepileptics *(N = 711)*		Antidepressants *(N = 657)*	
	Generic switch N (%)	No generic switch N (%)	Generic switch N (%)	No generic switch N (%)
	183 (25.7)	528 (74.3)	234 (35.6)	423 (64.4)
Earlier generic switching within the index ATC code				
None	18 (10.2)	326 (64.3)	39 (17.3)	163 (39.7)
≥1 switches	158 (89.8)	181 (35.7)	187 (82.7)	248 (60.3)
Gender				
Male	75 (41.0)	236 (44.7)	64 (27.4)	127 (30.0)
Female	108 (59.0)	292 (55.3)	170 (72.6	296 (70.0)
Age mean (SD)	54.2 (14.5)	56.4 (14.3)	54.4 (15.3)	55.9 (14.2)
Number of different drugs				
1 drug	23 (12.6)	60 (11.4)	36 (15.4)	62 (14.7)
2–4 drugs	73 (39.9)	202 (38.2)	109 (46.6)	173 (40.9)
≥5 drugs	87 (47.5)	266 (50.4)	89 (38.0)	188 (44.4)
Redemptions of the index drug within 1 year prior to index date				
2 redemptions	10 (5.4)	13 (2.5)	23 (9.8)	25 (5.9)
3–4 redemptions	13 (7.1)	55 (10.4)	63 (26.9)	95 (22.5)
5–6 redemptions	36 (19.7)	90 (17.1)	58 (24.8)	85 (20.1)
7–8 redemptions	38 (20.8)	118 (22.3)	34 (14.6)	92 (21.7)
≥9 redemptions	86 (47.0)	252 (47.7)	56 (23.9)	126 (29.8)
Concerns about medicine (BMQ)				
Low score (not concerned)	114 (62.3)	341 (64.6)	152 (65.0)	259 (61.2)
High score (concerned)	69 (38.7)	187 (35.4)	82 (35.0)	164 (38.8)
Views on generic medicine				
Positive	163 (89.1)	404 (76.5)	215 (91.9)	365 (86.3)
Negative	20 (10.9)	124 (23.5)	19 (8.1)	58 (13.7)

Data are presented as the number (of subjects), with the percentage given in parenthesis

**Fig 2:**
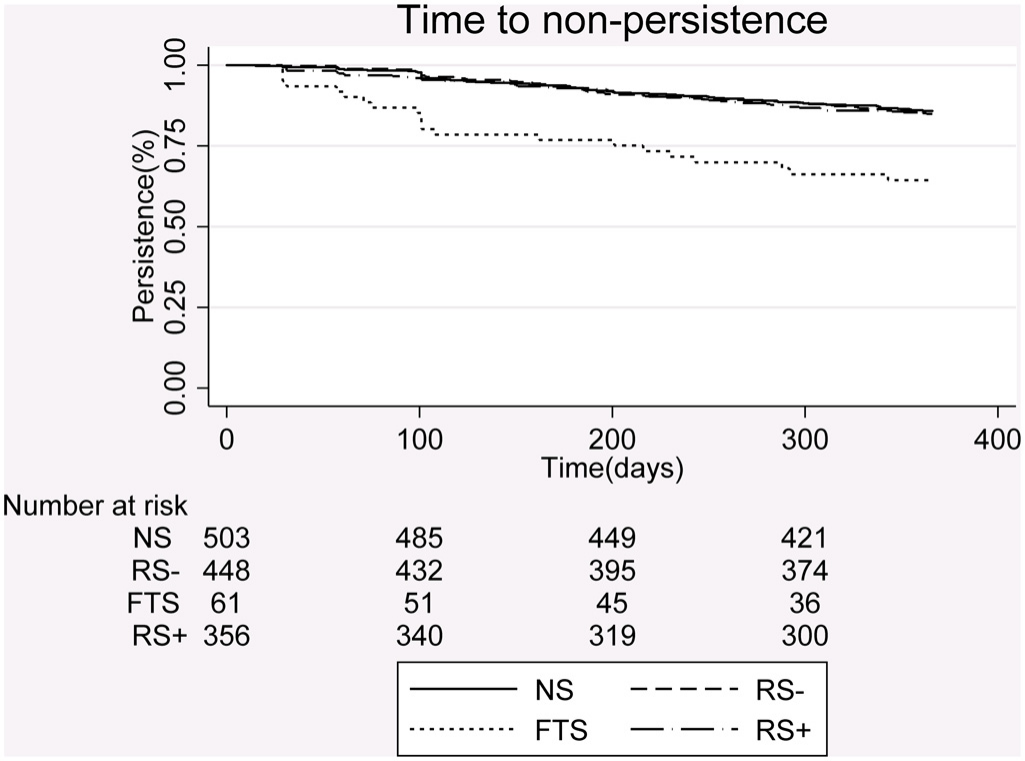
Kaplan-Meier plots of time to non-persistence having switched generics or not. **NS**: Never Switchers, **RS-**: Recurrent Switchers without generic switch on index day, **FTS**: First Time Switchers and **RS+**: Recurrent Switchers with generic switch on index day.

During the 365 days of follow-up 237 (17.3%) patients included in the study became non-persistent to their treatment (Fig. 1). Table 2 shows that patients who experience their first generic switch had a higher risk of non-persistence of the index drug over time; HR 2.98, 95% CI (1.81;4.89) compared to never switchers. Generic switching did not influence persistence considerably in those having previous experience with generic switching of the specific drug. Fig. 2 shows that the time to non-persistence differed according to the patients’ experience with generic switching. Among first-time switchers 35.7% became non-persistent during the first year of follow-up. In contrast, among patients who had never experienced a switch 14.2% became non-persistent. Among patients with previous experience with generic switching within the index ATC code, 15.0% became non-persistent if they switched on the index day and 15.1% if they did not switch on the index day.

**Table 2:** Hazard ratio of non-persistence.

		Univariate Hazard Ratio (95% CI)	Adjusted Hazard Ratio (95% CI)
Generic switch / earlier generic			
index date	switch index ATC		
No	No	1.00 (ref)	1.00 (ref)
No	Yes ≥1earlier switch	1.06 (0.76;1.49)	1.02 (0.72;1.43)
Yes	No	3.07 (1.88;5.00) ***	2.98 (1.81;4.89)***
Yes	Yes ≥1earlier switch	1.07 (0.74;1.53)	0.98 (0.68;1.41)
Gender			
Female		1.00 (ref)	1.00 (ref)
Male		0.98 (0.74;1.30)	1.02 (0.77;1.37)
Age (years)			
20–29		1.00 (ref)	1.00 (ref)
30–39		0.48 (0.24;0.96) *	0.51 (0.25;1.03)
40–49		0.58 (0.32;1.04)	0.58 (0.32;1.06)
50–59		0.43 (0.24;0.79) **	0.45 (0.24;0.82) **
60–69		0.63 (0.35;1.11)	0.66 (0.36;1.21)
70+		0.48 (0.26;0.89) *	0.49 (0.26;0.95) *
Number of different drugs			
1 drug		1.00 (ref)	1.00 (ref)
2–4 drugs		0.89 (0.59;1.36)	0.91 (0.60;1.39)
≥5 drugs		0.86 (0.57;1.29)	0.93 (0.60;1.43)
BMQ specific concerns^a^ (low score)		1.00	1.00
High score		1.47 (1.11;1.93)**	1.54 (1.17;2.03) **
Views on generic medicine^b^ (positive)		1.00	1.00
Negative		0.66 (0.43;1.01)	0.65 (0.42;0.99)*

The adjusted model: adjusted for gender, age, number of different drugs, BMQ concerns and Views on generic medicine.

*p<0.05,

**p<0.01 and

***p<0.001

^a^Table footnotes belong here: BMQ specific concerns assesses beliefs about the index drug—it represents beliefs about the danger of dependence and long-term toxicity and disruptive effects of medication

^b^Table footnotes belong here: Views on cheaper generic medicine compared to more expensive medicine in terms of side effects, quality and effectiveness

The Cox regression analyses were also performed stratified on drug categories, i.e. antidepressants and antiepileptics, both showing higher risk of non-persistence when the patients experienced their first generic switch of the index drug (Table not shown). The group of antidepressant users had a HR of 2.19, 95% CI (1.21;3.96) and the users of antiepileptics a HR of 2.89, 95% CI (1.09;7.69) for non-persistence among first-time switchers versus never switchers. Interaction between the two drug categories was tested and no interaction was found.

Other potential confounding variables in the model such as age, concerns about medicine and views on generics had an effect on persistence. However, it did not affect our primary predictor considerably. Sensitivity analysis assessing the influence of gap length did not materially affect the association between switching patterns and non-persistence (Table 3).

**Table 3:** Influence of length of grace period on estimated non-persistence.

		Length of grace period (days)			
Hazard ratio (95% CI)		30	60	90	120
Generic switch / earlier generic					
Index date	switch index ATC				
No	No	1 (ref)	1 (ref)	1.00 (ref)	1 (ref)
No	Yes ≥1 earlier switch	1.14 (0.86;1.50)	1.05 (0.77;1.45)	1.02 (0.72;1.43)	0.98 (0.68;1.41)
Yes	No	2.41 (1.55;3.74)***	2.81 (1.75;4.53)***	2.98 (1.81;4.89)***	2.67 (1.57;4.54)***
Yes	Yes ≥1 earlier switch	1.21 (0.91;1.62)	1.01 (0.72;1.43)	0.98 (0.68;1.41)	0.99 (0.67;1.44)

Hazard ratios between generic switch and non-persistence

Non-persistence was established as the first episode in a subjects’ medication history with a gap in prescription renewal that exceeded a predefined limit (number of tablets and a grace period of 90 days)

Hazard ratios are presented as the full model described in Table 2

*p<0.05,

**p<0.01 and

***p<0.001

## Discussion

### Summary

We found that patients who were first-time switchers of a specific drug were at higher risk of non-persistence versus never switchers or multiple switchers, respectively. The stratified analyses showed higher risk of non-persistence for first time switchers for both drug categories, i.e. antidepressants and antiepileptics.

### Strength and limitations

This study adds to the body of knowledge about the mechanisms of non-persistence in a wide group of patients, both addressing first-time switchers and recurrent switchers. A major strength of the study was that it by means of prescription data focused on a single well-defined generic switch, and that the purchase was confirmed by the patient in the questionnaire. Additionally we obtained information on previous generic switches on the same specific drug within one year. In that way we had a unique opportunity to look into patients’ overall experience with generic switch of one specific drug.

The prescription register data offered complete coverage on the use of reimbursed drugs by all cohort members [24]. Furthermore, we were able to combine the register data with questionnaire items on views on generic medicines and the validated concerns about medicine from the BMQ. The OPED prescription database did not have information on prescribed daily doses, which would have been the ideal measure for continuity calculations. However, as tablets come in all clinically relevant strengths, it is unlikely that patients take less than one tablet per day. For patients using more than one tablet per day we may have underestimated non-persistence.

The choice of non-persistence rather than non-adherence [29] was made because of our interest in whether patients stay on their therapy, when a generic switch has taken place. The definition of non-persistence with a 90-day grace period was based on literature [29,31,32]. For drugs such as antiepileptics and antidepressants missed doses may be more problematic and decrease the effectiveness of therapy compared to missed doses of other classes of drugs, e.g. antihypertensive agents, implying that a short grace period should be used [29]. However, the sensitivity analyses showed robustness of the results irrespective of the length of the grace periods with results having the same direction with narrow confidence intervals.

Regarding the questionnaire a possible selection due to the sampling procedure cannot be ruled out. The GPs were allowed to exclude vulnerable patients and excluded 6.4%. The response rate was 44.1%, which corresponds to other questionnaire survey studies [33]. Switchers and non-switchers were quite similar among respondents and non-respondents [14,15], and distribution of age, gender and drug group of the non-respondents was quite similar to the distribution of the sample, hence we assume that our results are generalizable to the population of drug users. Also, the relatively short interval between the drug purchase and receiving the questionnaire probably minimized the recall bias.

### Comparison with existing literature

There is consistency between this study’s results and previous studies comprising incident users, Ström et al. found that patients who had their medicine substituted at their first prescription refill had a higher probability of discontinuing treatment [7]. Kesselheim et al. also studied incident medication users, in this case users of anticonvulsants, and found that changes in pill color or shape due to generic substitution were associated with discontinuation [34]. The grace period employed was, however, only 5 days, which might have led to an overestimated rate of non-persistence. Studies pointing in other directions are e.g. Van Wijk et al. who assessed non-adherence among incident users of antihypertensive medicine, showing that generic substitution improved medication adherence, but a possible weakness of the study was a relatively short follow-up period of 180 days [6]. Olesen et al. assessed adherence and generic substitution in an elderly population with polypharmacy by means of pill count, and the results of that study also showed that generic substitution did not affect adherence negatively [9]. However, the indirect measure of adherence, that is pill count, has been found to overestimate adherence [35]. Persistence studies often measure the duration of time from initiation to discontinuation of therapy in incident drugs users or previous “treatment naïve” patients [7,34,36]. Studies evaluating incident users of therapy may report lower estimates of persistence than our study, representing patients with at least two prescriptions, since the largest non-persistence occurs within the first year of therapy [29]. What this study specifically shows is that first-time switching is the most critical point. Experience with generic switching has been shown to influence acceptance of future generic switches positively [14]. This study also shows that experience with generic switching also has a positive influence on persistence.

Concerns and cautions have been raised in relation to generic substitution of antidepressants and especially antiepileptics [37,38]. When looking at this study’s two drug categories, the persistence estimate had the same direction with different results, but with overlapping confidence intervals. The non-persistence estimate was higher among users of antiepileptics than among users of antidepressants. Generic substitution in the treatment of epilepsy has raised concerns at both patient and physician level. Despite the fact that anticonvulsants have narrow therapeutic indices, studies have shown that many physicians were likely to request brand antiepileptics “dispensed as written” because of concerns about breakthrough seizures [37,39]. Pechlivanoglou et al. showed that users of antidepressants were more prone to redeem brand name products [40].

It is well known that decisions about taking medication are likely to be influenced by beliefs about medicines as well as beliefs about the illness, and studies have reported negative associations between low adherence and specific concerns about medicine [27,41] and specifically for users of antiepileptics and antidepressants [42,43]. Results from the present study support these studies, showing that patients with a high level of concerns were negatively associated with persistence. Surprisingly, negative views on generics were positively associated with persistence. An explanation could be that patients having negative views on generics may have thought rationally about their medicine and over time chosen to take their medicine as prescribed. However, both questionnaire items did not affect our primary predictor considerably in the adjusted model.

In conclusion, patients who are first-time switchers of a specific drug were at higher risk of non-persistence compared to never switchers and those having experienced previous generic switch.

### Implication for research and practice

This study shows that experience with changes in medication due to generic substitution is of major importance and that first-time switchers need special attention, e.g. information from prescribing physicians or pharmacy professionals. It seems to be important to give words to potential changes to patients’ medicine both at physician consultations and at the pharmacies. Focus on the name of the active substance may be of some relevance to patients, which could give them a possibility to navigate by medication lists issued by physicians and by emphasizing the name of the active substance name on a sticker on the drug package, which was introduced in Denmark 2013. Hence interventions should be developed targeting this specific event, to support physicians, pharmacists and most importantly patients.

## Supporting Information

S1 Questionnaire Views on generic medicine. The ad hoc constructed scale applied in the questionnaire: “Views on generic medicine”.
(DOCX)
