# Supplementary material for: Generic Switching and Non-Persistence among Medicine Users: A Combined Population-Based Questionnaire and Register Study
Source: PLoS One. 2015 Mar 16;10(3):e0119688. doi: 10.1371/journal.pone.0119688 (PMC4361595; doi:10.1371/journal.pone.0119688)
Supplement: S1 Questionnaire — The ad hoc constructed scale applied in the questionnaire: “Views on generic medicine”. (DOCX) [file pone.0119688.s001.docx]

**Below you will find some statements on generic medicine. We would like to know whether you agree.**

16A. Inexpensive medicine works just as well as expensive medicine containing the same active ingredient

16B. Inexpensive medicine has more side effects than expensive medicine containing the same active ingredient

16C. Inexpensive medicine is of the same quality as expensive medicine containing the same active ingredient

16D. Medicine from two different manufacturers has same effect, when it contains the same active ingredient
